# RickA Expression Is Not Sufficient to Promote Actin-Based Motility of *Rickettsia raoultii*

**DOI:** 10.1371/journal.pone.0002582

**Published:** 2008-07-09

**Authors:** Premanand Balraj, Khalid El Karkouri, Guy Vestris, Leon Espinosa, Didier Raoult, Patricia Renesto

**Affiliations:** Unité des Rickettsies, URMITE IRD-CNRS UMR 6236, Faculté de Médecine, Marseille, France; Centre for DNA Fingerprinting and Diagnostics, India

## Abstract

**Background:**

*Rickettsia raoultii* is a novel *Rickettsia* species recently isolated from *Dermacentor* ticks and classified within the spotted fever group (SFG). The inability of *R. raoultii* to spread within L929 cells suggests that this bacterium is unable to polymerize host cell actin, a property exhibited by all SFG rickettsiae except *R. peacocki*. This result led us to investigate if RickA, the protein thought to generate actin nucleation, was expressed within this rickettsia species.

**Methodology/Principal Findings:**

Amplification and sequencing of *R. raoultii rickA* showed that this gene encoded a putative 565 amino acid protein highly homologous to those found in other rickettsiae. Using immunofluorescence assays, we determined that the motility pattern (i.e. microcolonies or cell-to-cell spreading) of *R. raoultii* was different depending on the host cell line in which the bacteria replicated. In contrast, under the same experimental conditions, *R. conorii* shares the same phenotype both in L929 and in Vero cells. Transmission electron microscopy analysis of infected cells showed that non-motile bacteria were free in the cytosol instead of enclosed in a vacuole. Moreover, western-blot analysis demonstrated that the defect of *R. raoultii* actin-based motility within L929 cells was not related to lower expression of RickA.

**Conclusion/Significance:**

These results, together with previously published data about *R. typhi*, strongly suggest that another factor, apart from RickA, may be involved with be responsible for actin-based motility in bacteria from the *Rickettsia* genus.

## Introduction

Rickettsiae are obligate intracellular Gram-negative bacteria that are associated with arthropod vectors and are responsible for mild to severe diseases in humans [1]. The bacteria from the *Rickettsia* genus are classified in three main groups: the *Rickettsia bellii* group including *R. bellii* and *R. canadensis*, the typhus group (TG), that is composed of *R. prowazekii* and *R. typhi*, and the spotted fever group (SFG), which encompasses more than 20 different species such as *R. conorii* and *R. rickettsii* [2]. For a long time, it was thought that members of the SFG rickettsiae differed from those of the TG in their capacity to promote directional actin polymerization [3], [4]. Accordingly, the rickettsial factor responsible for the motility of rickettsiae was identified through a comparative analysis of the *R. conorii* and *R. prowazekii* genomes [5]. This gene encodes for a protein called RickA [6], which has sequence similarity with the human WASP family of nucleation-promoting factors which have the capacity to activate Arp2/3 *in vitro* [6], [7]. While *rickA* was thought to be present within the last common ancestor of rickettsiae, it was lost by the TG rickettsiae over the course of evolution [2]. The capacity of rickettsiae to use the actin-based motility system for promoting cell-to-cell spreading was noticeable in several SFG rickettsiae including *R. conorii* and virulent and avirulent strains of *R. rickettsii*, *R. montanensis*, *R. parkeri*, *R. australis* and *R. monacensis* [8]–[10]. In contrast, *R. peacockii*, an SFG rickettsia that is closely related to *R. rickettsii*, was unable to form actin-tails [11], [12]. The lack of actin-based motility in *R. peacockii* was hypothesized to be a result of the disruption of *rickA* by an inserted sequence of 1,095 nucleotides termed IRSpe1 [13].

*Rickettsia raoultii* (*Rickettsia* sp. genotypes DnS14) was first identified as a new rickettsiae of the *R. massiliae* genogroup in 1999, by *rrs* (16S rDNA), *gltA* and *ompA* sequencing from *Dermacentor nutallii* ticks collected in Siberia [14]. The cultivation of rickettsial isolates genetically identical to *Rickettsia* sp. genotype DnS14 was recently achieved. From multi-gene sequencing, their classification within a new species called *Rickettsia raoultii* sp. nov. was proposed [15]. Preliminary microscopy observations showed that *R. raoultii* was apparently devoid of motility since the bacteria formed microcolonies in L929 in a similar way to *R. peacockii* [13] and *R. prowazekii* [16], [17]. Thus, we investigated the molecular basis accounting for such a phenotype, and began by sequencing *rickA*. The sequence of the predicted protein was then compared with the RickA protein of the other SFG rickettsiae. Further investigations highlighted a variable motility phenotype of *R. raoultii*, as assessed through immunofluorescent staining and plaque formation. Such a defect in actin-based motility occurred, and bacteria were able to escape the vacuole; this was unrelated to the expression level of RickA.

## Results

### Sequencing and phylogenetic analysis of *R. raoultii* RickA

**Figure 1:**
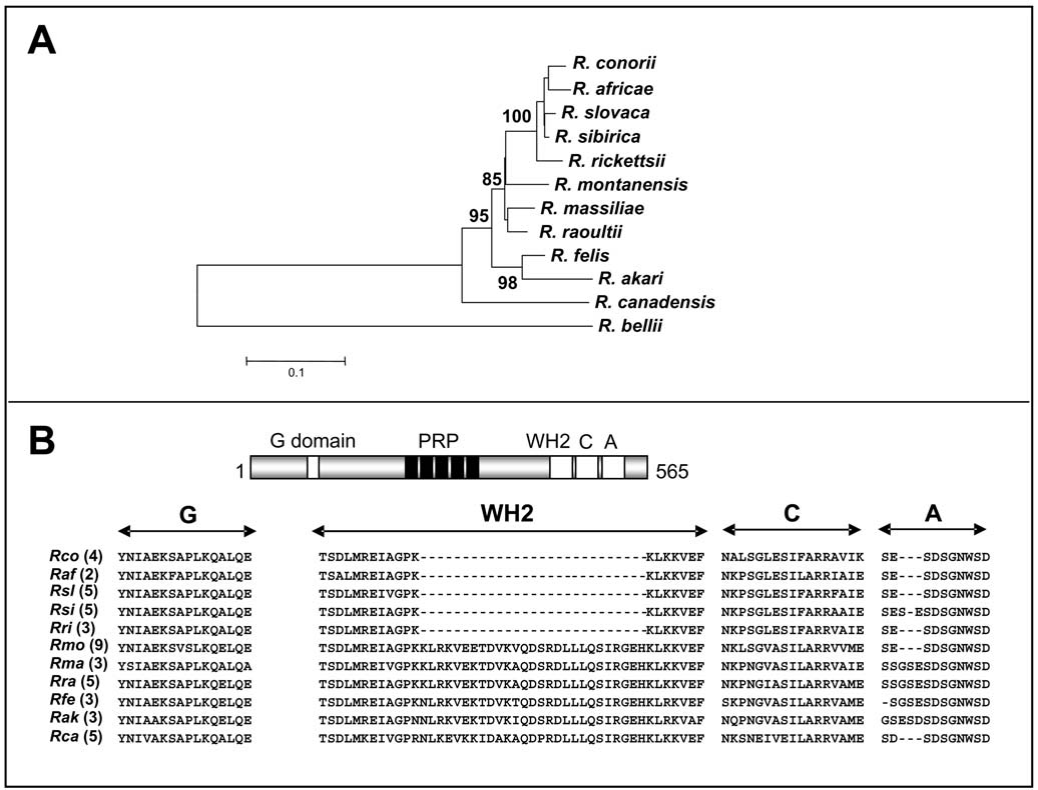
(A) Phylogenetic tree of the bacteria belonging to the genus *Rickettsia*, inferred from a comparison of RickA protein sequences. This phylogenetic tree was constructed with the NJ method, and using the following RickA protein sequences: *R. conorii* (rco_ORF0822), *R. rickettsii* (gi|157828764|), *R. slovaca* (RISLO1054), *R. africae* (raf_ORF0824) *R. sibirica* (ZP00142939), *R. massiliae* (gi|157844512|), *R. felis* (gi|67458763|), *R. akari* (gi|157825980|), *R. canadensis* (gi|157803553|), *R. bellii* (gi|91205670|), *R. montanensis* (gi|75487919|) and *R. raoultii* (this study). Bootstrap values are indicated at branch nodes. (B) Schematic representation and multiple sequence alignment of the rickettsial RickA proteins from the SFG rickettsiae. G: G-actin binding site. WH2, C and A: WASP-homology 2, central and acidic domains of the WASP-family protein. The numbers of proline-rich repeats (PRR) are indicated in parentheses. Abbrevations are as follows: *Rco* (*R. conorii*), *Raf* (*R. africae*), *Rsl* (*R. slovaca*), *Rsi* (*R. sibirica*), *Rri* (*R. rickettsii*), *Rmo* (*R. montanensis*), *Rma* (*R. massiliae*), *Rra* (*R. raoultii*), *Rfe* (*R. felis*), *Rak* (*R. akari*) and *Rca* (*R. canadensis*).

Using primers specific to the conserved regions flanking the *rickA* gene in several rickettsial genomes and with genomic *R. raoultii* DNA as the template, we successfully amplified a PCR fragment with a nucleotide size comparable to that obtained with *R. conorii*. Further sequencing of this amplicon indicated that the *rickA* gene of *R. raoultii* consisted of 1,695 basepairs (bp) coding for a 565 amino acid protein. A 44.2% to 89.6% level of identity was observed between the deduced amino acid sequence of the RickA protein from *R. raoultii* and the paralogous proteins present in the 11 other strains of rickettsiae included in this study (Table 1). *Rickettsia massiliae* exhibited the highest level of identity, the lowest was observed with *R. bellii*. The phylogenic trees inferred from the RickA data set and using either the maximum parsimony (MP) or neighbor-joining (NJ) method exhibited quite similar topologies (not shown). The RickA NJ tree distinguished two main phylogenetic clades with confident bootstrap values (Fig. 1A). Clade I encompassed *R. raoultii* with all *rickettsia* species examined, except for *R. felis*, *R. akari*, *R. canadensis* and *R. bellii*, with the bootstrap support (BP) 85%. Within this group, *R. raoultii* was found to be closely related to *R. massiliae*. Clade II contained *R. felis* and *R. akari* (BP = 98%). *R. canadensis* was placed into a single external branch while *R. bellii* appeared as an outgroup.

**Table 1:** Pairwise identity square matrix between RickA proteins from various rickettsia species.

Species	Size (aa)	*Raf*	*Rco*	*Rsl*	*Rsi*	*Rri*	*Rma*	*Rra*	*Rak*	*Rfe*	*Rmo*	*Rca*	*Rbe*
***R africae***	500	100	91.2	91.5	90.6	89.2	84.1	81.1	74.9	79.9	71.3	68.0	44.7
***R. conorii***	520		100	92.6	94.0	90.0	81.9	83.8	72.7	77.9	74.8	69.0	44.6
***R. slovaca***	516			100	96.4	91.3	82.7	85.0	74.0	78.3	75.0	71.0	46.3
***R. sibirica***	526				100	90.5	81.5	87.3	73.4	77.8	76.8	71.3	45.4
***R. rickettsii***	494					100	84.3	80.9	76.7	80.7	72.0	67.8	44.5
***R. massiliae***	532						100	89.6	83.7	90.1	76.8	72.1	44.0
***R. raoultii***	565							100	79.4	85.0	82.7	76.1	44.2
***R. akari***	523								100	88.9	70.1	67.7	43.1
***R. felis***	529									100	73.5	71.7	44.3
***R. montanensis***	602										100	68.5	41.8
***R. canadensis***	559											100	42.1
***R. bellii***	518												100

Values were deduced from the pairwise comparison of RickA sequences between different strains of rickettsiae. The abbreviations across the top correspond to the species on the left, from top to bottom, respectively.

### RickA is conserved in *R. raoultii*

The overall organization of RickA [6], [7] was found to be conserved within these 12 rickettsia species. Specifically, RickA contained an N-terminal domain for binding monomeric actin (G domain), a central proline-rich region believed to play a role in binding to WASP proteins and the WCA region including WASP-homology 2 (WH2), central (C) and acidic (A) domains that should interact with the Arp2/3-complex (Fig. S1). Within each of these regions, we observed that RickA proteins from the SFG rickettsiae underwent some changes (Fig. 1B). In the G domain, we mainly noticed that the alanine (A) found in *R. conorii*, *R. africae*, *R. slovaca*, *R. sibirica*, *R. rickettsii*, *R. massiliae* and *R. canadensis* was replaced by a glutamic acid (E) in other strains, including *R. raoultii*. Five proline-rich repeats (P), starting with the sequence motif [EGQKD]-N-N-[IM] were numbered in the RickA protein of *R. raoultii*. These values ranged from 2 (*R. africae*) up to 9 (*R. montanensis*) in other SFG rickettsiae. As initially described for *R. montanensis* [7], *R. raoultii* has two WH2 domains; this is also the case for *R. massiliae*, *R. akari*, *R. felis* and *R. canadensis*. While the C domain of *R. raoultii* RickA appeared highly similar (>88%) with those of *R. massilae* and *R. felis*, their A domains exhibited 100% homology with each other. As described in other rickettsiae [6], we failed to find evidence for a signal sequence at the N-terminus of *R. raoultii* RickA.

### The presence of *R. raoultii* actin tails varies according to the host eukaryotic cell line

**Figure 2:**
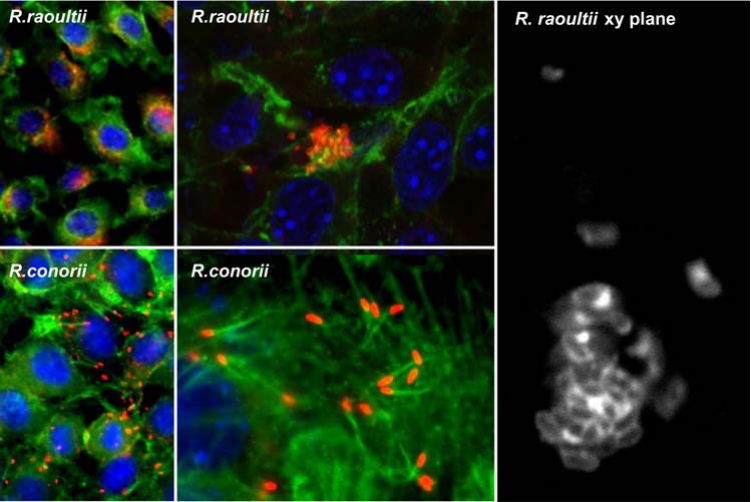
Actin tail phenotypes of *R. raoultii* and *R. conorii* in L929 cells. Following a 24 h infection, the bacteria were stained by indirect immunofluorescence using a polyclonal anti-SFG antibody followed by an anti-mouse-Alexa 594 Ig as a secondary antibody (red). F-actin was stained with FITC-phalloidin (green) and nucleic acids were stained with DAPI (blue). Examination of slides with a Leica DM2500 Upright fluorescent microscope (magnifications 40× and 100×), showed that *R. raoultii* formed microcolonies and multiplied at the center of the cell while *R. conorii* exhibited actin tails at one pole of the bacterium. The right panel corresponds to a higher-magnification image of *R. raoultii* microcolony observed with a Leica SPE upright confocal microscope.

**Figure 3:**
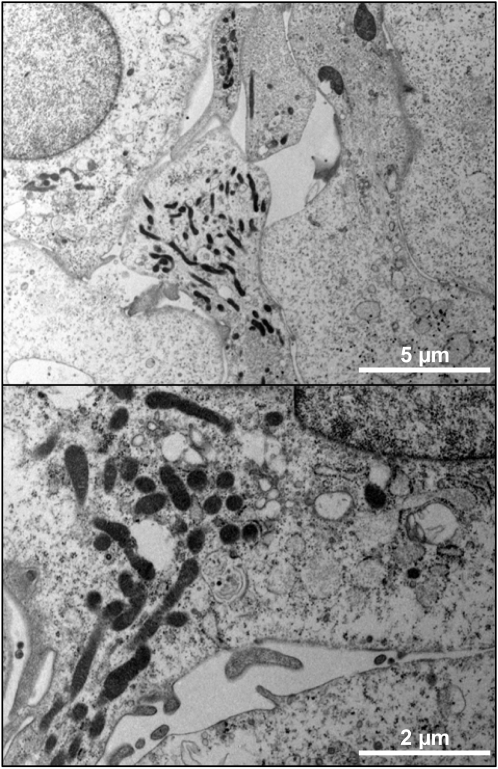
TEM of L929 cells infected with *R. raoultii*. (A) TEM performed on *R. raoultii* cultured for 48 h in L929 cells confirmed that bacteria multiply without infecting neighboring cells. (B) A higher magnification showed free bacteria in the cytoplasm and failed to demonstrate the presence of a membrane vacuole surrounding bacteria.

**Figure 4:**
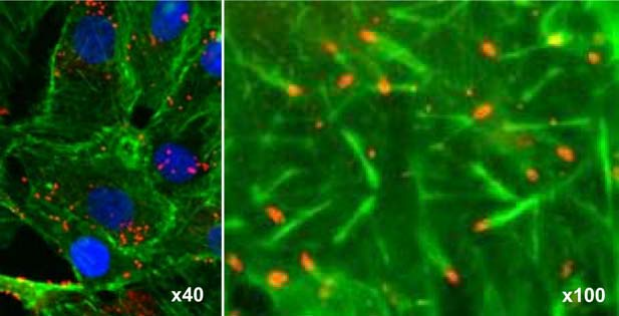
Actin tail phenotype of *R. raoultii* in Vero cells. The same staining as that described in the Fig. 2 was applied on Vero cells infected for 24 h with *R. raoultii* (magnifications 40× and 100×).

**Figure 5:**
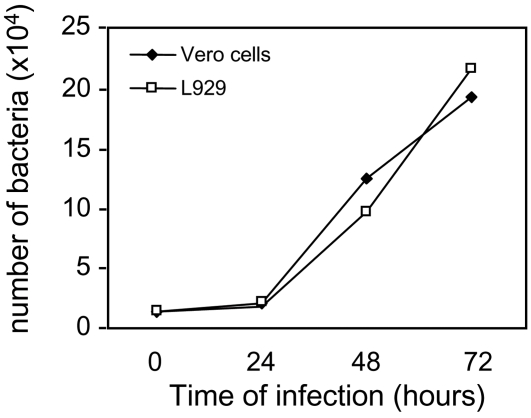
Intracellular growth of *R. raoultii*. Bacterial growth was evaluated by qRT-PCR as described in the Materials and Methods, using either L929 cells (black circles) or Vero cells (white circles). Eukaryotic cells approaching confluence and grown in shell vials were infected with 1.5×10^4^ bacteria. Each point corresponds to the mean of two distinct experiments.

**Figure 6:**
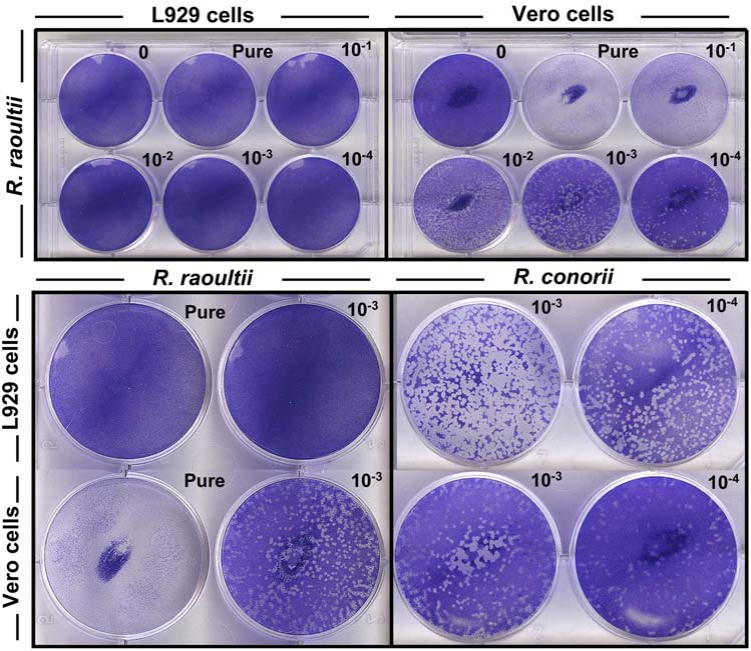
Plaque formation by *R. raoultii* and *R. conorii* in different cell lines. L929 or Vero cells monolayers were infected for 10 days with serial dilutions of bacteria, the pure inoculum being estimated by qRT-PCR as 2.5×10^7^ rickettsiae per well. The “control” corresponds to non-infected cells. Upper panel: After crystal violet-staining we noticed the absence of plaque formation in L929 cells infected with *R. raoultii* (left) while a concentration-dependent effect was observed on Vero cells (right). Lower panel: Magnification of the effects observed with *R. raoultii* or with *R. conorii*.

As illustrated in Fig. 2, when grown in L929 cells, *R. raoultii* appears as microcolonies. Under these experimental conditions, and despite several attempts, we failed to observe actin tail formation at the pole of the bacteria (not shown). Examination of infected cells by transmission electron microscopy demonstrated that rickettsiae were free in the cytoplasm (Fig. 3). When grown in Vero cells, these bacteria appeared more evenly distributed (Fig. 4A), a point consistent with the frequent detection of actin comets (Fig. 4B). The apparent lack of *R. raoultii* motility within L929 cells was further confirmed by plaque assays. Thus, while real-time quantitative PCR (qRT-PCR) measurements showed that the replication of *R. raoultii* was similar in both cell lines, (Fig. 5) the L929 monolayer was not at all damaged by *R. raoultii* (Fig. 6, upper left panel). In contrast, a concentration-dependent effect was observed in Vero cells where large plaques were observed (Fig. 6, upper right panel). By comparison, *R. conorii* induced plaque formation in both cell lines (Fig. 6, lower right panel). The size and the number of plaques were slightly higher in L929 than in Vero cells, but this difference was strictly correlated with a higher replication of bacteria within the former (not shown).

### The expression of RickA was similar in host eukaryotic cell lines

**Figure 7:**
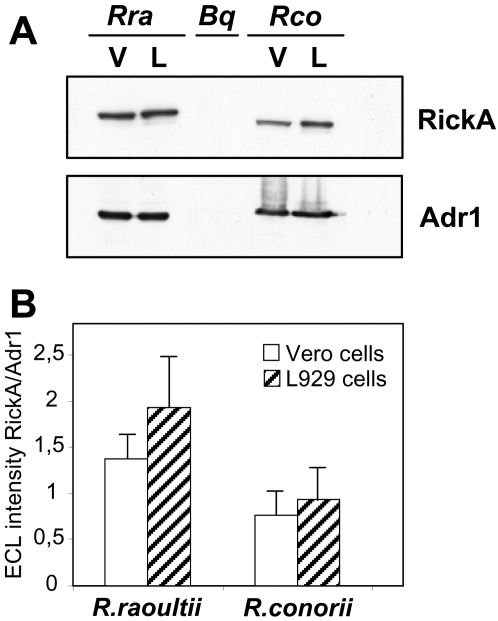
RickA expression of rickettsiae grown in different cell lines. (A) Vero cells (V) or L929 cells (L) infected with *R. raoultii* (*Rra*) or *R. conorii* (*Rco*) were subjected to SDS-PAGE and then transferred to a nitrocellulose membrane. The membranes were subsequently probed with anti-RickA or anti-Adr1 antibodies. Identical amounts of proteins were loaded in each lane. *Bartonella quintana* (*Bq*) whole extracts were used as a negative control. (B) Quantification of RickA expression normalized to Adr1 expression for each sample. Data are mean+/−SEM for three distinct experiments.

Western blot analyses were conducted on crude extracts of cells infected with rickettsiae, using a monoclonal anti-RickA antibody. The putative rickettsial adhesin encoded by RC1281 in the *R. conorii* genome, also called Adr1 [18], was used as a control. The quantification of the signal intensity for each band demonstrated that comparable amounts of RickA were present in *R. raoultii* grown in either L929 or Vero cells (*p*<0.05, Fig. 7). The same profile was observed with *R. conorii*.

### Comparative analysis of *R. prowazekii* and *R. typhi* genomes

Based on the recent comparative analysis of seven rickettsial genomes (http://www.igs.cnrs-mrs.fr/mgdb/Rickettsia/rig/data/RIG_Table.xls) [2], we compiled a list of genes present in *R. typhi* and absent or degraded in *R. prowazekii* (Table 2). Nineteen genes were identified, and the majority were conserved within all rickettsial genomes, except for the non-motile *R. prowazekii* strain.

**Table 2:** Rickettsial genes conserved in *R. typhi* and degraded in *R. prowazekii*.

RIG	B	F	M	C	A	P	T	gene	description
**RIG0687**	1	1	1	1	1	0,5	1	*gltD*	NADPH-dependent glutamate synthase beta chain and related oxidoreductases
**RIG0672**	0,5	1	1	1	1	0,5	1	*sca1*	Cell surface antigen Sca1
**RIG0660**	0	1	1	1	1	0,5	1		Putative methyltransferase
**RIG1174**	1	0,5	1	1	1	0,5	1		Unknown
**RIG1137**	1	0,5	0,5	1	1	0,5	1		Thermostable carboxypeptidase
**RIG1130**	1	1	1	1	1	0,5	1		Heat shock protease [EC:3.4.21.-]
**RIG0899**	1	1	1	1	1	0,5	1	*pbpC*	Bifunctional penicillin-binding protein 1C
**RIG1249**	1	1	1	1	1	0,5	1		Unknown
**RIG1278**	1	1	1	1	1	0,5	1	*msbA1*	Multidrug resistance protein
**RIG0576**	1	1	1	1	1	0,5	1	*recO*	DNA repair protein RecO
**RIG0571**	1	1	1	1	1	0,5	1		Unknown
**RIG0559**	1	1	1	1	1	0,5	1		Large extracellular alpha-helical protein
**RIG0552**	1	1	1	1	1	0,5	1	*ubiH*	2-polyprenyl-6-methoxyphenol 4-hydroxylase [EC:1.14.13.-]
**RIG0184**	0	1	0	0	1	0,1	1		Unknown
**RIG0791**	1	1	1	1	1	0,5	1	*ubiB*	2-polyprenylphenol 6-hydroxylase [EC:1.14.13.-]
**RIG0045**	1	1	1	0,5	1	0,5	1	*spoT11*, *spoTd*	Guanosine polyphosphate pyrophosphohydrolase/synthetase
**RIG0643**	1	1	1	1	1	0,5	1		Unknown
**RIG0617**	0	0,5	0,5	0,5	0,5	0,5	1	*metK*	S-adenosylmethionine synthetase
**RIG1579**	0	0,1	0	0	0	0,5	1	*sca6*	Cell surface antigen Sca6

B (*R. bellii*), F (*R. felis*), M (*R. massiliae*), C (*R. conorii*), A (*R. africae*), P (*R. prowazekii*), T (*R. typhi*).

full-length state = 1; pseudogene = 0.5 (for split genes) or 0.1 (for gene remnants); absent = 0.

## Discussion

We have sequenced the *rickA* gene of the recently isolated SFG rickettsiae, *R. raoultii* [15]. This gene encodes a protein of 565 amino acids, consistent with the length of other published RickA sequences, which vary from 494 aa for *R. rickettsii* up to 602 aa for *R. montanensis*. The dendrograms inferred from the comparison of available RickA sequences with two distinct tree-building analysis methods showed similar organization. We identified two main groups within representatives of the SFG: *R. akari* and *R. felis* clustered together, while another group consisted of *R. raoultii*, *R. africae*, *R. slovaca*, *R. sibirica*, *R. rickettsii*, *R. montanensis* and *R. massiliae*. The architecture of the tree was similar to that described using concatenated nucleotide sequences of the 16S rDNA, *gltA*, *pheS*, *pheT*, *nusA*, *valS* and *smpB* genes [19]. From our RickA-based phylogenetic analysis, we confirmed that the bacterium most closely related to *R. raoultii* was *R. massiliae* [15]. Within this tree, the rickettsiae that have the capacity to promote actin tail formation, *R. conorii* [8], [9], [20], *R. rickettsii* [9], *R. montanensis* [9], *R. felis* [21] and *R. bellii* [19], exhibited an overall distribution and did not cluster together. This observation suggests that the actin-based motility is a property that should be conserved within all rickettsia species encoding RickA.

The predicted structure of the RickA protein from *R. raoultii* shared the same organization as that of other SFG rickettsiae with highly conserved domains that are thought to be of functional significance. The amino acid sequence of the G-actin-binding site was found to be 100% homologous between *R. raoultii* and *R. felis*, and minor changes were observed with other rickettsiae. This result suggests that *R. raoultii* RickA should bind actin filaments as efficiently as the homologous protein present in *R. felis*, an SFG endowed with the capacity to promote actin tail formation [21]. Other RickA-specific regions located within the C-terminal end of the protein were also strongly conserved, including the proline-rich domains as well as the WASP-homology 2 (WH2), central (C), and acidic (A) domains believed to mediate the Arp2/3 complex activation.

We then examined if the absence of actin comets and cell-to-cell spreading observed when *R. raoultii* replicated in L929 cells was related with a defect in vacuole escape. This process is known to involve phospholipase D (PLD) [22], [23] and hemolysin C [23]. Analysis of the *R. raoultii* genome, in progress in our laboratory (unpublished data), revealed that both genes encode highly conserved proteins (Figs. S2 and S3). These data, as well as the absence of a vacuole membrane surrounding the bacteria, which nevertheless appeared to group within the cytosol of L929 cells, confirmed that the non-motile phenotype was related to the defect in actin tail-formation.

To clarify whether *R. raoultii* RickA failed to promote actin nucleation, thus leading to the formation of microcolonies, we performed immunofluorescent microscopy analysis of bacteria grown in different cell lines. Surprisingly, and in contrast to what was observed in the fibroblast cell line, when grown in the African green monkey kidney epithelial cells (Vero cell line), *R. raoultii* exhibited cell-to-cell spreading consistent with the detection of actin tails behind the bacteria. These results were further confirmed by plaque assays. Plaque formation, which quantifies the invasive capacity of bacteria, is a complex process involving several key factors such as efficiency of bacteria entry, lysis of the vacuole of phagocytosis, intracellular motility allowing cell-to-cell spreading, as well as the multiplication rate of bacteria and the time of infection. For the bacteria from the *Rickettsia* genus, it was previously reported that plaque formation could be related to the conditions employed, including centrifugation of inoculated monolayers, culture media and incubation temperature [24]–[26]. These findings are consistent with the relationship that might exist between such parameters and the efficiency of entry and replication. A difference in susceptibility between different cell lines was also observed [25]–[27]. It was thus demonstrated that *R. canadensis* produced plaques in Vero cells and not in primary cultures of chicken embryos, with the reverse being true for *R. prowazekii* 15GP/2EP strain [25]. Similarly, *R. rickettsii* failed to form plaques in HeLa and Hep-2 cultures, while plaques were observed in other cell lines tested [27]. It was reported that the limited capacity of rickettsiae to promote plaque formation in some cell lines was not correlated with a reduced replication [27]. Such a phenotype, commonly observed for the TG rickettsiae, was rather associated with the lack of membrane lytic factors or with a lower cell-to-cell spreading [28]. Here, when infected with *R. raoultii* under strictly similar experimental conditions, Vero cells exhibited plaque formation while the L929 cell monolayer remained intact. As previously reported for *R. rickettsii*, *R. parkeri* and *R. montanensis* [27], the growth of *R. raoultii* was found to be identical in whichever host cell line was used. These results, together with the variable actin tail phenotype exhibited by *R. raoultii* and the preserved ability of bacteria to escape the vacuole in L929, led us to establish a correlation between the absence of plaques in these cells and the observed defect of actin-based motility. This conclusion is also supported by the fact that plaque formation was induced to a similar extent in L929 and in Vero cells with *R. conorii*, a bacterium able to promote actin polymerization in both cell lines.

Because cell-to-cell spreading was lost in *icsA* (*Shigella flexneri*) [29], *actA* (Listeria monocytogenes) [30] and *bimA* (*Burkholderia pseudomallei*) [31] mutants, we examined whether the lack of actin tail formation observed for *R. raoultii* was accompanied with a reduced level of RickA. In contrast to what was expected, our results showed that the expression of this protein was similar when *R. raoultii* replicated in L929 or in Vero cells. The same profile was observed with *R. conorii*, and the size and morphology of plaques were nearly identical in both cell lines. These results indicated that RickA expression was not a sufficient condition to promote actin polymerization *in vivo*. It could be hypothesized that RickA is not in a functional state when *R. raoultii* replicate within L929. While it was still located on the bacterial surface [6], the mechanisms for RickA secretion, anchoring to the bacterial surface, and its putative polarized distribution remain to to be defined [3], [32]. Consequently, it is difficult to investigate at which stage a potential functional deficiency would take place. Alternatively, our data reinforce the scenario in which another factor, not yet identified, is involved in rickettsia cell-to-cell spreading [4]. While actin-based motility had long been confined to the SFG rickettsiae, the presence of small and rare actin tails was depicted for *R. typhi* [8], [9]. A recent time-lapse video microscopy study revealed that despite erratic movements, *R. typhi* moved at approximately the same rate as *R. rickettsii* [28]. Such motility displayed by a *Rickettsia* species that lacks *rickA* [33] suggests that another rickettsial protein can promote actin polymerization. The availability of genome sequences for rickettsiae exhibiting divergent motility phenotypes would be helpful in identifying other key bacterial players involved in such a process. Here, the comparative genomic analysis of the non-motile *R. prowazekii* with the motile *R. typhi*, which are both devoid of *rickA*, allowed for the identification of 19 genes which are present in the latter and degraded or absent in the former. Therefore, much more work will be required to confirm the putative roles of these proteins as actin nucleation factors. Because the transcriptional pattern of several intracellular pathogens exhibit host-cell specific features [34], we may hypothesize that the second candidate is differentially regulated by the host cell. The recently developed strategy allowing transcriptional analysis of rickettsiae would be helpful for investigating such a possibility. The host cells could thus play an indirect role by modulating the expression of bacterial proteins or by triggering post-translational modifications on RickA proteins or on other proteins influencing rickettsia motility. A relationship has indeed been established between the phosphorylation of the Acta protein of *Listeria monocytogenes* and the motility of this bacterium [35]. The host cell could also intervene as an active player by providing a factor that would facilitate bacterial motility as evoked about the stathmin recruitment by *L. monocytogenes* [36].

In summary, while RickA was recently classified as a virulence factor playing a role in bacterial exit [37], this work clearly demonstrates that RickA expression was not sufficient to promote *R. raoultii* motility. These results, together with the motile phenotype displayed by *R. typhi* [28] confirm that the picture of rickettsial motility is more complex than initially thought.

## Materials and Methods

### Bacterial strains and eukaryotic cell lines

The rickettsial strains used in the present study were *R. conorii* strain seven (ATCC, VR613), *R. raoultii* Khabarovsk^T^ ( = CSUR R3^T^) [15], *R. prowazekii* strain BreinL (ATCC VR-142T) and *R. typhi* strain Wilmington (ATCC-VR-144T). Rickettsiae were propagated within confluent monolayers of African green monkey kidney cells (Vero cell, ATCC C1587) or in the murine fibrobast L929 cell line (ATCC CCL 1), both maintained in Eagle's minimum essential medium (MEM, Gibco, Invitrogen, Paisley, UK) supplemented with fetal calf serum (FCS, Gibco) and L-glutamine (Gibco) as described [38]. Infections were achieved from frozen rickettsiae previously purified on a sucrose gradient [18] and stored in glycerol. By qRT-PCR, the concentration of the stock solution of bacteria was determined and adjusted to 5×10^7^/ml. Before each experiment, the degree of infection was estimated by Gimenez staining. In western-blot assays, *Bartonella quintana* (strain Fuller, ATCC VR-358^T^) was used as the negative control. Protein concentration was measured by BioRad DC assay (BioRad, Hercules, CA, USA).

### PCR amplification and sequencing of the *rickA* gene and flanking DNA

DNA was extracted from Vero cells infected with rickettsiae using a QIAamp DNA mini kit (Qiagen, Hilden, Germany), according to the manufacturer's instructions. Primers used to amplify *R. raoultii rickA* had been designed based on the alignment of the *rickA* sequences of *R. conorii* (GB AE008644), *R. montanensis* (GB AJ293315), *R. felis* (GB CP000053) and *R. rickettsii* (GB AJ293314). These oligonucleotides were numbered from the *R. conorii rickA* sequence. The whole *R. raoultii rickA* amplicon was obtained with primers RIC−30F and RIC+740R. Primer RIC−30F was designed in the *mraY-rickA* spacer (−30 bp upstream) and RIC+740R in the *rick A*- *wrbA* (tryptophan repressor binding protein) spacer (+11 bp downstream). The PCR amplifications were performed with a PE 9600 thermal cycler (Applied Biosystems, Courtaboeuf, France) using the Expand High Fidelity PCR system (Roche Diagnostics, Meylan, France). The following conditions were used for amplification: pre-denaturation for 5 min at 95°C, followed by 39 cycles of denaturation for 30 s at 94°C, annealing for 45 s at 55°C, and extension for 2 min at 68°C. Amplification was completed by holding the reaction mixture for 5 min at 72°C. Resulting PCR products were purified using a QIAquick Spin PCR purification kit (Qiagen). They were then sequenced in both directions with specific primers and a D-rhodamine Terminator cycle sequencing ready reaction kit (Applied Biosystems). Sequencing products were resolved using an ABI 3100 automated sequencer (Perkin-Elmer, Saint-Quentin-en-Yvelines, France). The primers used in this study are summarized in Table S1.

### Nucleotide sequence accession number

The sequence of *R. raoultii rickA* has been deposited in GenBank with accession number n°EU340900.

### Comparative analysis of RickA proteins

The sequences of the RickA proteins from 12 *Rickettsia* species, obtained from GenBank (http://www.ncbi.nlm.nih.gov/), RickBase (http://igs-server.cnrs-mrs.fr/mgdb/Rickettsia/) and our current work were used for phylogenetic inference. In a first step, a pairwise alignment of these sequences was carried out using the needle algorithm of emboss suite (http://www.ebi.ac.uk/emboss/align/index.html), thus allowing for identity determination between the 12 RickA proteins. In a second step, a multiple sequence alignment was performed using the clustalw algorithm of the bioedit software with default settings, and then manually corrected [39], [40]. Both neighbor joining (NJ) and maximum parsimony (MP) trees were constructed using the MEGA 3.1 software [41]. Branch robustness was estimated through bootstrap analysis of 1,000 and 100 replicates for NJ and MP trees, respectively [42].

### Western-blot assay

Infected cells were harvested using 3 mm diameter glass beads, and the cell suspension was centrifuged at 7,500×g for 10 min. The pellets were resuspended in PBS, and the protein concentration was estimated with the Bradford method before solubilizing in Laemmli buffer. Samples (20 µg/well) separated using 10% sodium dodecyl sulfate-polyacrylamide gel electrophoresis (SDS-PAGE) were transferred to a nitrocellulose membrane (Amersham, Buckinghamshire, UK). Western-blot analysis was performed using either a monoclonal antibody against the recombinant RickA protein (1∶1,000) or a mouse polyclonal antibody (1∶5,000) produced in the laboratory, against the Adr1 protein encoded by RC1281 in *R. conorii* (unpublished data). After washing, the membranes were incubated with anti-mouse or anti-rabbit secondary antibodies conjugated to HRP (Amersham) and detected using chemiluminescence (ECL system, Amersham). Quantification of western-blots was performed on scanned images. The intensities of the bands recognized with anti-RickA mAbs were normalized to Adr1 expression

### Immunofluorescent staining of actin tails

Eukaryotic cells grown to semiconfluence on glass coverslips were infected with *R. raoultii* (1.5×10^4^ bacteria/shell-vial) for 24 h and fixed for 1 h at ambient temperature with paraformaldehyde (3% w/v in phosphate-buffered saline (PBS) supplemented with 1 mM MgCl_2_ and 1 mM CaCl_2_). Following gentle washing with PBS, the cells were permeabilized with 0.1% Triton X-100 in PBS for 30 sec before incubation for 45 min in humidified chamber with a rabbit anti-*R. conorii* antibody (1∶1,000). After washing with PBS, the samples were incubated with an anti-rabbit Alexa 546 (1∶300; Molecular probes, Invitrogen, Cergy-Pontoise, France) secondary antibody and with FITC-phalloidin (1∶300; Sigma-Aldrich, Saint Quentin Fallavier, France) for actin staining. After three further washings, the coverslips were air-dried and mounted with DAPI (4′,6-diamidino-2-phenylindole) from a ready-to-use solution, ProLong Gold Antifade Reagent (Molecular Probes). Images were acquired on a Leica DM2500 Upright fluorescent microscope with Cooled DS1-QM (Nikon, Champigny sur Marne, France) black and white camera and Lucia G acquisition software (LIM Ltd. Prague, Czech Republic). Confocal images were obtained with a Leica SPE upright confocal microscope with 63×N.A. = 1.4 objective and the thickness of optical slides was set to 0.3 microns. The fluorescent components (phalloidin, Alexa 546 and DAPI) were acquired sequentially.

### Electron microscopy

Transmission electron microscopy (TEM) analysis was conducted on L929 cells infected with *R. raoultii*. A 125 cm^2^-flask infected with 10^7^ bacteria for 48 h was carefully collected and pelleted by centrifugation before fixation for 1 h at room temperature in 2.5% glutaraldehyde (Electron Microscopy Sciences, Hatfield, PA, USA) in 0.1 M phosphate buffer for 4 h. Samples were further fixed for 1 h at room temperature with 1% osmium tetroxide, dehydrated through increasing concentrations (25 to 100%) of ethanol and embedded in Epon 812 resin (Electron Microscopy Sciences). Thin sections (70 nm) were cut and poststained with 4% uranyl acetate and lead citrate in water before examination on a Philips Morgagni 268 D electron microscope (FEI Compagny France, Limeil-Brevannes, France).

### Plaque assays

Vero cells or L929 cells were seeded in six-well plates (Greiner bio-one, CELLSTAR) and incubated at 37°C under a 5% CO_2_ atmosphere in 1.5 ml of MEM supplemented with 4% FCS and 2 mM glutamine. After overnight growth, the medium was removed, and 500 µl of rickettsiae diluted in the same medium were added to the monolayers. The cells were subsequently incubated at 37°C for 60 minutes. The inocula were ranging between 2.5×10^7^ and 2.5×10^3^ bacteria/well. During this attachment phase, the plates were rocked every 30 min to ensure uniform distribution of bacteria. The overlay medium mixture (3.5 ml) consisting of culture medium supplemented with 0.5% agar (Sigma) was then cooled to 42°C and added to each well. The plates were incubated at 37°C for 10 days to watch for plaque formation. The agar-based overlays were then gently removed using a spatula, and monolayers were fixed with 3% formaldehyde before crystal violet staining.

### Real-time quantitative PCR

Determination of 16S rRNA gene copy numbers was performed using the probes 5′ TGATGAAGGCCTTAGGGTTG 3′ and 5′ TAAACCGCCTACGCACTCTT 3′ on the LightCycler system together with the SYBR Green master mix (Roche). DNA extracted from eukaryotic cells grown in shell-vials and infected by *R. conorii* or *R. raoultii* (QIAamp DNA mini kit, Qiagen, Courtaboeuf, France) was used as a template. The yield of qRT-PCR product was assessed by plotting the cycle threshold values versus the log_10_ numbers of input DNA copies. The DNA content of each sample was determined using a standard curve obtained by parallel processing of samples containing titrated DNA concentrations. Each qRT-PCR assay was done in duplicate starting from at least two independent experiments.

## Supporting Information

Figure S1 Alignement of RickA proteins from various rickettsiae.
(0.53 MB DOC)

Figure S2 Alignement of R. conorii and R. raoultii PLD.
(0.03 MB DOC)

Figure S3 Alignement of R. conorii and R. raoultii Hemolysin C.
(0.04 MB DOC)

Table S1 Oligonucleotide primers used for PCR amplification and sequencing of rickA.
(0.03 MB DOC)

## References

[1] Raoult D, Roux V (1997). Rickettsioses as paradigms of new or emerging infectious diseases.. Clin Microbiol Rev.

[2] Blanc G, Ogata H, Robert C, Audic S, Suhre K (2007). Reductive genome evolution from the mother of *Rickettsia*.. PloS Genet.

[3] Gouin E, Welch MD, Cossart P (2005). Actin-based motility of intracellular pathogens.. Curr Opin Microbiol.

[4] Stevens JM, Galyov EE, Stevens MP (2006). Actin-dependent movement of bacterial pathogens.. Nat Rev Microbiol.

[5] Ogata H, Audic S, Renesto-Audiffren P, Fournier PE, Barbe V (2001). Mechanisms of evolution in *Rickettsia conorii* and *R. prowazekii*.. Science.

[6] Gouin E, Egile C, Dehoux P, Villiers V, Adams J (2004). The RickA protein of *Rickettsia conorii* activates the Arp2/3 complex.. Nature.

[7] Jeng RL, Goley ED, D'Alessio JA, Chaga OY, Svitkina TM (2004). A Rickettsia WASP-like protein activates the Arp2/3 complex and mediates actin-based motility.. Cell Microbiol.

[8] Teysseire N, Chiche-Portiche C, Raoult D (1992). Intracellular movements of *Rickettsia conorii* and *R. typhi* based on actin polymerization.. Res Microbiol.

[9] Heinzen RA, Hayes SF, Peacock MG, Hackstadt T (1993). Directional actin polymerization associated with spotted fever group Rickettsia infection of Vero cells.. Infect Immun.

[10] Baldridge GD, Burkhardt N, Herron MJ, Kurtti TJ, Munderloh UG (2005). Analysis of fluorescent protein expression in transformants of *Rickettsia monacensis*, an obligate intracellular tick symbiont.. Appl Environ Microbiol.

[11] Simser JA, Palmer AT, Munderloh UG, Kurtti TJ (1999). Isolation and characterization of *Rickettsia peacockii* maintained in tick cell culture. *Am J Trop Med Hyg*
**61**(Suppl.): 359.. Am J Trop Med Hyg.

[12] Baldridge GD, Burkhardt NY, Simser JA, Kurtti TJ, Munderloh UG (2004). Sequence and expression analysis of the *ompA* gene of *Rickettsia peacockii*, an endosymbiont of the Rocky Mountain wood tick, *Dermacentor andersoni*.. Appl Environ Microbiol.

[13] Simser JA, Rahman MS, Dreher-Lesnick SM, Azad AF (2005). A novel and naturally occurring transposon, ISRpe1 in the *Rickettsia peacockii* genome disrupting the *rickA* gene involved in actin-based motility.. Mol Microbiol.

[14] Rydkina E, Roux V, Rudakov N, Gafarova M, Tarasevich I (1999). New Rickettsiae in ticks collected in territories of the former soviet union.. Emerg Infect Dis.

[15] Mediannikov O, Matsumoto K, Samoylenko I, Drancourt M, Roux V (2008). *Rickettsia raoultii* sp. nov., a new spotted fever group rickettsia associated with *Dermacentor* ticks in Europe and Russia.. Int J Syst Evol Microbiol.

[16] Smirnova NS, Popov VL, Kokorin IN (1986). Peculiarities of *Rickettsia prowazekii* in the cell culture as revealed by cryoultramicrotomy.. Acta Virol.

[17] Wisseman CLJ, Waddell AD (1975). In vitro studies on rickettsia-host cell interactions: intracellular growth cycle of virulent and attenuated *Rickettsia prowazeki* in chicken embryo cells in slide chamber cultures.. Infect Immun.

[18] Renesto P, Samson L, Ogata H, Azza S, Fourquet P (2006). Identification of two putative rickettsial adhesins by proteomic analysis.. Res Microbiol.

[19] Ogata H, La Scola B, Audic S, Renesto P, Blanc G (2006). Genome sequence of Rickettsia bellii illuminates the role of amoebae in gene exchanges between intracellular pathogens..

[20] Gouin E, Gantelet H, Egile C, Lasa I, Ohayon H (1999). A comparative study of the actin-based motilities of the pathogenic bacteria *Listeria monocytogenes*, *Shigella flexneri* and *Rickettsia conorii*.. J Cell Sci.

[21] Ogata H, Renesto P, Audic S, Robert C, Blanc G (2005). The Genome Sequence of *Rickettsia felis* Identifies the First Putative Conjugative Plasmid in an Obligate Intracellular Parasite.. PLoS Biology.

[22] Renesto P, Dehoux P, Gouin E, Touqui L, Cossart P (2003). Identification and characterization of a phospholipase D-superfamily gene in rickettsiae.. J Infect Dis.

[23] Whitworth T, Popov VL, Yu XJ, Walker DH, Bouyer DH (2005). Expression of the *Rickettsia prowazekii* pld or tlyC gene in *Salmonella enterica* serovar Typhimurium mediates phagosomal escape.. Infect Immun.

[24] Wike DA, Tallent G, Peacock MG, Ormsbee RA (1972). Studies of the rickettsial plaque assay technique.. Infect Immun.

[25] Cory J, Yunker CE, Ormsbee RA, Peacock M, Meibos H (1974). Plaque assay of rickettsiae in a mammalian cell line.. Applied Microbiology.

[26] Osterman JV, Parr RP (1974). Plaque Formation by *Rickettsia conori* in WI-38, DBS-FRhL-2, L-929, HeLa, and Chicken Embryo Cells.. Infect Immun.

[27] Johnson JW, Pedersen CE (1978). Plaque formation by strains of spotted fever rickettsiae in monolayer cultures of various cell types.. J Clin Microbiol.

[28] Heinzen RA (2003). Rickettsial Actin-Based Motility: Behavior and Involvement of Cytoskeletal Regulators.. Ann N Y Acad Sci.

[29] Bernardini ML, Mounier J, d'Hauteville H, Coquis-Rondon M, Sansonetti PJ (1989). Identification of icsA, a plasmid locus of Shigella flexneri that governs bacterial intra- and intercellular spread through interaction with F-actin.. Proc Natl Acad Sci U S A.

[30] Kocks C, Gouin E, Tabouret M, Berche P, Ohayon H (1992). *L. monocytogenes*-induced actin assembly requires the actA gene product, a surface protein.. Cell.

[31] Stevens MP, Stevens JM, Jeng RL, Taylor LA, Wood MW (2005). Identification of a bacterial factor required for actin-based motility of *Burkholderia pseudomallei*.. Mol Microbiol.

[32] Carlsson F, Brown EJ (2006). Actin-based motility of intracellular bacteria, and polarized surface distribution of the bacterial effector molecules.. J Cell Physiol.

[33] McLeod MP, Qin X, Karpathy SE, Gioia J, Highlander SK (2004). Complete genome sequence of *Rickettsia typhi* and comparison with sequences of other rickettsiae.. J Bacteriol.

[34] La MV, Francois P, Rovery C, Robineau S, Barbry P (2007). Development of a method for recovering rickettsial RNA from infected cells to analyze gene expression profiling of obligate intracellular bacteria.. J Microbiol Methods.

[35] Roberts AJ, Wiedmann M (2006). Allelic exchange and site-directed mutagenesis probe the contribution of ActA amino-acid variability to phosphorylation and virulence-associated phenotypes among Listeria monocytogenes strains.. FEMS Microbiol Lett.

[36] Yoshida S, Handa Y, Suzuki T, Ogawa M, Suzuki M (2006). Microtubule-severing activity of *Shigella* is pivotal for intercellular spreading.. Science.

[37] Hybiske K, Stephens RS (2008). Exit strategies of intracellular pathogens.. Nat Rev Microbiol.

[38] Roux V, Raoult D (1997). Genotypic identification and phylogenetic analysis of the spotted fever group rickettsiae by pulsed-field gel electrophoresis.. J Bacteriol.

[39] Thompson JD, Higgins DG, Gibson TJ (1994). CLUSTAL W: improving the sensitivity of progressive multiple sequence alignment through sequence weighting, position-specific gap penalties and weight matrix choice 200.. Nucleic Acids Res.

[40] Hall TA (1999). BioEdit: a user-friendly biological sequence alignment editor and analysis program for Windows 95/98/NT.. Nucl Acids Symp Ser.

[41] Kumar S, Tamura K, Nei M (2004). MEGA3: Integrated software for Molecular Evolutionary Genetics Analysis and sequence alignment.. Brief Bioinform.

[42] Felsenstein J (1985). Confidence limits on phylogenies with a molecular clocks.. Syst Zool.

