# Supplementary material for: RickA Expression Is Not Sufficient to Promote Actin-Based Motility of Rickettsia raoultii
Source: PLoS One. 2008 Jul 9;3(7):e2582. doi: 10.1371/journal.pone.0002582 (PMC2440523; doi:10.1371/journal.pone.0002582)
Supplement: Figure S1 — Alignement of RickA proteins from various rickettsiae. (0.53 MB DOC) [file pone.0002582.s001.doc]

                                         10        20        30        40        50        60           
                                ....|....|....|....|....|....|....|....|....|....|....|....|
R.conorii                       ---------VYGMVKEIDINKLLAQENNALNTILSQVNELCKQNKQLQGLIEIQNETKEL 
R.slovaca                       ------------MVKEIDINKLLAQENNALNTILSQVNELCKQNKQLQGLIEIQNETKEL 
R.africae                       ---------MYGMVKEIDINKLLAQENNALNTILSQVNELCKQNKQLQGLIEIQNETKEL 
R.sibirica                      ------------MVKEIDINKLLAQENNALNTILSQVNELCKQNKQLQGLIEIQNETKEL 
R.rickettsii                    ------------MVKEIDINKLLAQENNALNAILSHVNELCKQNKQLQGLIEIQNETKEL 
R.massiliae                     ---------MYGVAKEIDINKLLAQENNALNTILSQVNELCEQNKQLQGLIEIQNETKEL 
R.montanensis      		  ------------MTKEIDINKLLAQENNALNTILSQVNELCEQNKQLQGLIEIQNETKAL 
R.raoultii                      ---------VYGVAKEIDINKLLAQENNALNTILSQVNELCEQNKQLQGLIEIQNETKEL 
R.felis                         ---------MYGVAKEIDINKLLAQENNALNTILSQVNELCEQNKKLQGLIEIQNETKEL 
R.akari                         ------------MAQEIDINKLLAQENNALNAILSQVNELCTQNKKLQGLIEIQNKTKEL 
R.canadensis                    MAKETKETKETKETKETDINKLLTQENNALNTILRQVNELCEQNQKLQGVLEIQNEAKEL 
R.bellii                        ------------MAKITELDHHLNQEKEALDKVVSNLNELCEHNQKLQGFIEIQKEVKEL 

                                         70        80        90       100       110       120        
                                ....|....|....|....|....|....|....|....|....|....|....|....|
R.conorii                       EKEHNRSLPWFKRFVKTVSNVKYILIKSEEQLTNEAIKYNNKILKDIDNKIYNIAEKSAP 
R.slovaca                       EKEHNRSLPWFKRFVKTVSNVKYILIKSEEQLTNEAIKYNNKILKDIDNKIYNIAEKSAP 
R.africae                       EKEHNRSLPWFKRFVKTVSNVKYILIKSEEQLTNEAIKYNNRILKDIDNKIYNIAEKFAP 
R.sibirica                      EKEHNRSLPWFKRFVKTVSNVKYILIKSEEQLTNEAIKYNNKILKDIDNKIYNIAEKSAP 
R.rickettsii                    EKEHNRSLPWFKRFVKTVSNVKYILIKSEEQLTNEAIKYNNKILKDIDNKIYNIAEKSAP 
R.massiliae                     EKEHNRSLPWFKRLVKTVSNVKYIFIKSEEQLTNEAIKYNNKILKDIDNKIYSIAEKSAP 
R.montanensis      		  EKEYNRSLPWFKRFVNTVSNVKYIFIKSEEQLTNEAIKYNNKILKDIDNKIYNIAEKSVS 
R.raoultii                      EKEHNRSLPWFKRFVKTVSNVKYIFIKSEEQLTNEAIKYNNKILKDIDNKIYNIAEKSAP 
R.felis                         EKEHNRSLPWFKRLVKTVSNVKYIFVKSEEQLTNEAIKYNNKILKDIDNKIYNIAEKSAP 
R.akari                         AKEHNRSLPWFKRLVKTVSNVKYIFVKSEEQLTNEAIKYNNKVLKSIDNKIYNIAAKSAP 
R.canadensis                    EKEHNRSLPWFKKFVKTVSNVKYIFIKSEEQLTHEAIKHNTKILKDIDNKIYNIVAKSAP 
R.bellii                        KKEHIKSLSWFKKLINTVSNIKYVFVKSEEQLAKDAIEQNNKLLKRIDNTILSVADKSGP 

                                        130       140       150       160       170       180     
                                ....|....|....|....|....|....|....|....|....|....|....|....|
R.conorii                       LKQALQEEIEKNFKDLTKKDLSKDQRARLSEVF-FSYKSKPERFSALHMTNPLQFINAEA 
R.slovaca                       LKQALQEEIEKNFKDLTKKDLSKDQRARLSEVF-FSYKSKPERFSALHMTNPLQFINAEA 
R.africae                       LKQALQEEIEKNFKDLTKKDLSKDQRARLSEVF-FSYKSKPERFSALHMTNPLQFINAEA 
R.sibirica                      LKQALQEEIEKNFKDLTKKDLSKDQRARLSEVF-FSYKSKPERFSALHMTNPLQFINAEA 
R.rickettsii                    LKQALQEEIEKSFKDLTKKDLSKDQRARLSEVF-FSYKSKPERFSALHMTNPLQFINAEA 
R.massiliae                     LKQALQAEIEKIFKDLTKKDLSKDQRARVSEVF-FSYKSKSERFSALHMTNPLQFINAEE 
R.montanensis      		  LKQELQEEIEKNFKDLTKKDLSKDQRERLSEVF-FSYKSKPERFSALHMTNPLQFINAEE 
R.raoultii                      LKQELQEEIEKNFKDLTKKDLSKDQRERLSEVF-FSYKSKPERFSALHMTDPLQFINAEE 
R.felis                         LKQELQEEIEKNFKDLTKKDLSKEQRERLSEVY-FSYKSKPERFSALNMTNPLQFIKAEE 
R.akari                         LKQELQEEIAKNFKDLTKKDLSKEQRERLSEVY-FSYKSKPERFSALHITNPLQFINAEE 
R.canadensis                    LKQALQEEIKKNFEDLTKKDLSKDQRERLAELF-FSYKDKPERFSALRMTHPLQFTNAAV 
R.bellii                        LKQELQKELRKNFENLAKKDLSKDQRERLSNLLNNEYAANPQKFAQLPMSKPLHFPNAEE 

                                        190       200       210       220       230       240     
                                ....|....|....|....|....|....|....|....|....|....|....|....|
R.conorii                       LEKQYNSLNATKQNIQNLISANSNIKELKEIQKQVAEIRAEVPHTFFEKLNNIWQNVKNV 
R.slovaca                       LEKQYNALNATKQNIQNLISENSNIKELKEIQKQVAEIRAEVPHTFFEKLNNIWQNVKNV 
R.africae                       LEKQYNALNATKQNIQNLISENSNIKELKEIQKQVAEIRAEVPHTFFEKLNNIWQNVKNV 
R.sibirica                      LEKQYNSLNATKQNIQNLISENSNIKELKEIQKQVAEIRAEVPHTFFEKLNNIWQNVKNV 
R.rickettsii                    LEKQFNSLNATKQNIQNLISENSNIKELKEIQKQVAEIRAEVPHTFFEKLNNIWQNVKNV 
R.massiliae                     LEKQYNSLNATKQNIQNLISENSNIKELKEIQKQVAEIREEVPYTFFEKLNNIWQNVKNV 
R.montanensis      		  LEKQYNSLNATKQNIQNLISENSNVKELKEIQKQVAEIREEVPYTFFEKLNNIWQNVKNV 
R.raoultii                      LEKQYNSLNATKQNIQNLISENSNIKELKEIQKQVAEIRAEVPYTFFEKLNNIWQNIKNV 
R.felis                         LEKQYNSLNATKQNIQNLISENSNIKELKEIQKQVAEIREEIPYTFFEKLNNIWQNVKNV 
R.akari                         LEKQYNSLNATRQNIQNLISENSNIKELKEIQKQVAEIREEIPYTFFEKLNNIWQNVKNV 
R.canadensis                    LENQYNALNTTKQNIRNLISENSNIKELKEIQKQVAEIREEIPHTFFEKLNSIWQNVKNV 
R.bellii                        LENQHNDLKVIQQNVLNLLTENSNIEELKKIQKQVAEIREEVPFTKLEKLNNFWQKIKNI 

                                        250       260       270       280       290       300     
                                ....|....|....|....|....|....|....|....|....|....|....|....|
R.conorii                       FVNNSEQVLAKNKESNTRTIRKIDEQLYKTKHKFEELIENKERNIKDIIAKLPDNEKLQK 
R.slovaca                       FVNNSEQVLAKNKESNTRTIRKIDEQLYKTKHKFEELIENKERNIKDIIAKLPDNEKLQK 
R.africae                       FVNNSEQVLAKNKESNTRTIRKIDEQLYKTKHKFEELIENKERNIKDIIAKLPDNEKLQK 
R.sibirica                      FVNNSEQVLAKNKESNTRTIRKIDEQLYKTKHKFEELIENKERNIKDIIAKLPDNEKLQK 
R.rickettsii                    FVNNSEQVLAKNKESNTRTIRKIDEQLYKTQHKFEELIENKERNIKDIIAKLPDNEELQK 
R.massiliae                     FVNNSEQVLAKNKESNTRTIRKIDEQLYKTKHKFEELIENKERNINDIIAKLPDNEELQK 
R.montanensis                   FVNNSEQVLAKNKESNTRTIRKIDEQLYKTKHKFEELIENKERNINDIIAKLPDNEELQK 
R.raoultii                      FVNNSEQVLAKNKESNTRTISKIDEQLYKTKHKFEELIENKERNINDIIAKLPDNEELQK 
R.felis                         FVNNSEQVLAKNKESNTRAIRKIDEQLYKTKHKFEELIENKERNINDIIAKLPDNEELQK 
R.akari                         FVNNKEKILAKNKESNTRTIRKIDEQLYKTKHKFEELIENKERNINDIISKLPDNEELKN 
R.canadensis                    FINNSEQVLAKNKESNTRAIRKIEEQLYSTKHQFEKLIENQEKNINDIIGKLPDNEKLQK 
R.bellii                        FVNNSEQVLAKNKENNTKTIINIEEKLHKANNKFFELVSNKKQDIENIISNLPDSKRLEA 

                                        310       320       330       340       350       360     
                                ....|....|....|....|....|....|....|....|....|....|....|....|
R.conorii                       IVSNLTNHMASQKEPILANASLAKPLENNITPPSP---LPENNIPSP----------PPP 
R.slovaca                       IVSNLTNHMASKKEPILANASLAKPLENNITPPPP---LPENNIP------------PPP 
R.africae                       IVSNLTNHMTSQKEPILANASLAKPLENNITPPSP---LSENNIPPS----------PPP 
R.sibirica                      IVSNLTNHMASQKEPILANASLAKPLENNITPPSP---LPENNIPSP----------PPP 
R.rickettsii                    IVSNLTNHMASKKEPILANVSLAKPLENNIPPP------------------------PPP 
R.massiliae                     IASNLTNHMASKKEPILANSSLAKPLENNITPPPP---LSQNNIPSP----------PPP 
R.montanensis                   IVSNLTNHMASTKEPILTNSSLAKPLENNITPPPP---LPGNNIPSP----------PPP 
R.raoultii                      IVSNLTNHMALKKEPILANSSLAKPLENNITPPPP---LSQNNIPSP----------PPP 
R.felis                         IVSNLANHMTSKKEPILTTSSIAKPLENNVTPPPP---LTKNNIP-------------PP 
R.akari                         IVSNFANHMTSKKEPILTTSSIAKPLENNITRPPL---LSQNNIP-------------SP 
R.canadensis                    IVSNLADHMTAKKEPILTTSSLTKPLENNMPPPPPPPPLPQNNMP-P----------PPP 
R.bellii                        IKEKLQKHINVKDTNNIAEQASAAQLQSAETKPTA-VVLPNNAIPTTPPVTEEKTFTPPP 

                                        370       380       390       400       410       420     
                                ....|....|....|....|....|....|....|....|....|....|....|....|
R.conorii                       PPPSPLPENNIPSSPPPPPPPPLPENNIPSPPPPP------------------------- 
R.slovaca                       PPP--LPENNIP--PPPPPPLPENNIP--PPPPPPLPENNIP------------------ 
R.africae                       PPP-----------PPPPPPP---------PPSPP------------------------- 
R.sibirica                      PPP--LPENNIPSPPPPPPPLPENNIPSPPPPPPPLPENNIPS----------------- 
R.rickettsii                    PPP--LPDSNIP--PPPPPPLPGNNIP--------------------------------- 
R.massiliae                     PPP--LPENNIP------------------------------------------------ 
R.montanensis                   PPP--LPGNNIPSPPPPPPPLPGNNIPSPPPPPPPLPGNNIPSPPPPPPPLSGNNIPSPP 
R.raoultii                      PPP--LPENNIPSPPPPPPPLPENNIPSPPPPPPPLPENNIPSPP--------------- 
R.felis                         PPPPPLSKNNIL-----PP----------------------------------------- 
R.akari                         P---PLSKNNIP-----PP----------------------------------------- 
R.canadensis                    PPP--LPQNNMP-PPPPPPPLPQNNMPPPPPPPPPLPQNN-------------------- 
R.bellii                        APPPPMPTDNIPTPLPVSKAEATEHKNVETAASN-------------------------- 

                                        430       440       450       460       470       480     
                                ....|....|....|....|....|....|....|....|....|....|....|....|
R.conorii                       -------------PPPPPPPMA------------------------------PAQAETLS 
R.slovaca                       -------------PPPPPPPPP------------------------------MAQAETLS 
R.africae                       -------------PPPPPPPMA------------------------------PAQAETLS 
R.sibirica                      ------------PPPPPPPPPP------------------------------MAPAETLS 
R.rickettsii                    -------------PPPPPPPPP------------------------------MAPVKTLS 
R.massiliae                     ------------PPPPPPPPMPT-------------------------MAPVSAQTETLS 
R.montanensis                   PPPPPLSGNNIPSPPPPPPPLPGNNIPSPPPPPPPLSQNNIPPPPPPPMAPVSAQTEKLS 
R.raoultii                      ------------PPPPPPPPMPT-------------------------MAPASAQTETLS 
R.felis                         -------------PPPPMPTMA------------------------------PAQTETLS 
R.akari                         -------------PPPPPPPMP------------------------------TAQTDALS 
R.canadensis                    -------------MPPPPPPMPT---------------------------MAPAQTETLL 
R.bellii                        ------------VPPPPPPPMPTG--------------------------NVPPPPPVGD 

                                        490       500       510       520       530       540     
                                ....|....|....|....|....|....|....|....|....|....|....|....|
R.conorii                       KPIESTT-VKKLANQPRPSIDTSDLMREIAGPK--------------------------- 
R.slovaca                       KPVESTT-VKKLENQPRPSIDTSDLMREIVGPK--------------------------- 
R.africae                       KPVESTT-VKKLENQPRPSIDTSALMREIAGPK--------------------------- 
R.sibirica                      KPVESTT-VKKLENQPRPSIDTSDLMREIAGPK--------------------------- 
R.rickettsii                    KAVEATT-VKKLENQPRPSIDTSDLMREIAGPK--------------------------- 
R.massiliae                     KPVEATT-VKKPENQLRPSIDTSDLMREIVGPKKLRKVEKTDVKAQDSRDLLLQSIRGEH 
R.montanensis                    KPVEATT-VKKPENQPRPSIDTSDLMREIAGPKKLRKVEETDVKVQDSRDLLLQSIRGEH 
R.raoultii                      KPVEATT-VKKPENQPCPSIDTSDLMREIAGPKKLRKVEKTDVKAQDSRDLLLQSIRGEH 
R.felis                         KPVGVTTTVKKLENQPRPSIDTSDLMREIAGPKNLRKVEKTDVKTQDSRDLLLQSIRGEH 
R.akari                         KPVGVTT-VKKLENQQRPSLDTSDLMREIAGPNNLRKVEKTDVKIQDSRDLLLQSIRGEH 
R.canadensis                    KSIEATT-VKNPENQSHPSIDTSDLMKEIVGPRNLKEVKKIDAKAQDPRDLLLQSIRGEH 
R.bellii                        NTVTSTPQKAKETNQPRPAVDTTNLMKQIQGGFN-------------------------- 

                                        550       560       570       580       590       600     
                                ....|....|....|....|....|....|....|....|....|....|....|....|
R.conorii                       KLKKVEFDPNTGKPVAHSHSKPAQNVNALSGLESIFARRAVIKVSD-SSSSE---SDSGN 
R.slovaca                       KLKKVEFDPNTGKPVTHSHSKPAQNVNKPSGLESIFARRFAIEVSD-SSSSE---SDSGN 
R.africae                       KLKKVEFDPNTGKPVVHSHSQPAQDVNKPSGLESILARRIAIEVSD-SSSSE---SDSGN 
R.sibirica                      KLKKVEFDPNTGKPVAHSHSKPAQNVNKPSGLESIFARRAAIEVSD-SSSSES-ESDSGN 
R.rickettsii                    KLKKVEFDPNTGKPVAHSHSKPAQNVNKPSGLESIFARRVAIEMSD-SSSSE---SDSGN 
R.massiliae                     KLKKVEFDPNTGKPVAHSHSKPAQNINKPNGVASILARRVAIEMSD-SSSSSGSESDSGN 
R.montanensis                   KLKKVEFDPNTGKPVAHSHSKPVQNVNKLSGVASILARRVVMEMSD-SSGSE---SDSGN 
R.raoultii                      KLKKVEFDPNTGKPVAHSHSKPAQNVNKPNGIASILARRVAMEMSD-SSSSSGSESDSGN 
R.felis                         KLRKVEFDPNTGKPVAHSHSKPAQNVSKPNGVASILARRVAMEMSDSSSS-SGSESDSGN 
R.akari                         KLRKVAFDPNTGKPVAHSHSKPAKNVNQPNGVASILARRVAMEMSDSSSSGSESDSDSGN 
R.canadensis                    KLKKVEFDPN-GRPVAHSRSKPAQNVNKSNEIVEILARRVAMEMSSSGSESD---SDSGN 
R.bellii                        -LKKIEYGED-GKPIPKNKEDTKETSDP--IIAALNKIRSAKVSSDSERSNSDSGTDSGW 

                                        610       620       630       640         
                                ....|....|....|....|....|....|....|....|....|
R.conorii                       WSDVSVNRNKSKMLKTKGERDAKMTTHAQK-INNRNSQNPSFVR- 
R.slovaca                       WSDASVNRNKSKMLKTKGERDAKMTTHAQK-INNRNSQKPSIVR- 
R.africae                       WSDVSVNRNKSKVLKTKGERDATMTTHAQK-INNRNSQKPSLVR- 
R.sibirica                      WSDVSVNRNKSKMLKTKGERDAKMTTHAQK-INNRNSQKPSFVR- 
R.rickettsii                    WSDVSVNRNKSKMLKTKGERDAKMTTHAQK-INNRNSQKPSFVR- 
R.massiliae                     WSDVSVNRNKSKSLKTKGERDAKMTTHAQKILNNRHSQKPSFVR- 
R.montanensis                   WSDVGVNRN-TKTLKTKRER--------RKILNNRNSQKPSFVK- 
R.raoultii                      WSDVSVNRNKSKILKTKGERDAKMTTHAQKTLNHRNSQKPSFVR- 
R.felis                         WSDASVNSNKPKALKTRGERDAKTTTHAQKILSNRSSQKPSFVRS 
R.akari                         WSDISVNSDKPKALKNRRERDGKRTTHAQKILSNRSSQKPSFVRS 
R.canadensis                    WSDVS---TKSKVLKTKGERDARKGMNSKQ--VGRNS-KSSFVRS 
R.bellii                        ASDVS---TRSKKVLTRRERNAKQSQQR----------------- 
