# Supplementary material for: RickA Expression Is Not Sufficient to Promote Actin-Based Motility of Rickettsia raoultii
Source: PLoS One. 2008 Jul 9;3(7):e2582. doi: 10.1371/journal.pone.0002582 (PMC2440523; doi:10.1371/journal.pone.0002582)
Supplement: Figure S2 — Alignement of R. conorii and R. raoultii PLD. (0.03 MB DOC) [file pone.0002582.s002.doc]

Figure S2- Alignement of R. conorii and R. raoultii PLD
Program: needle, Align format: srspair, Identity 198/200 (99%)


                                         10        20        30        40        50        60           
                                ....|....|....|....|....|....|....|....|....|....|....|....|
rco_ORF1154 3505084408 RC1270   MKRKNNKFIEISIAFILGIALGLYGQNPDYFTNLISQKSLALSALQIKHYNISELSRSKV 
C109RRA0006 PLD R. raoultii     MKRKNNKFIEISIAFILGIALGIYGQNPDYFTNLISQKSLALSALQIKHYNISELSRSKV 

                                         70        80        90       100       110       120        
                                ....|....|....|....|....|....|....|....|....|....|....|....|
rco_ORF1154 3505084408 RC1270   STCFTPPAGCTKFIANQIDKAEESIYMQAYGMSDALITTALINAQARGVKVRILLDRSNL 
C109RRA0006 PLD R. raoultii     STCFTPPAGCTKFIANQIDKAEESIYMQAYGMSDALITTALINAQARGVKVRILLDRSNL 

                                        130       140       150       160       170       180     
                                ....|....|....|....|....|....|....|....|....|....|....|....|
rco_ORF1154 3505084408 RC1270   KQKFSKLHELQRAKIDVDIDKVPGIAHNKVIIIDKKKVITGSFNFTAAADKRNAENVIII 
C109RRA0006 PLD R. raoultii     KQKFSKLHELQRAKIDVGIDKVPGIAHNKVIIIDKKKVITGSFNFTAAADKRNAENVIII 

                                        190       200 
                                ....|....|....|....|
rco_ORF1154 3505084408 RC1270   EDQELAESYLQNWLNRKASN 
C109RRA0006 PLD R. raoultii     EDQELAESYLQNWLNRKASN 
