# Supplementary material for: RickA Expression Is Not Sufficient to Promote Actin-Based Motility of Rickettsia raoultii
Source: PLoS One. 2008 Jul 9;3(7):e2582. doi: 10.1371/journal.pone.0002582 (PMC2440523; doi:10.1371/journal.pone.0002582)
Supplement: Figure S3 — Alignement of R. conorii and R. raoultii Hemolysin C. (0.04 MB DOC) [file pone.0002582.s003.doc]

Figure S3- Alignement of R. conorii and R. raoultii Hemolysin C
Program: needle, Align format: srspair, Identity 291/299 (97.3%)

                                         10        20        30        40        50        60           
                                ....|....|....|....|....|....|....|....|....|....|....|....|
rco_ORF1032 2890083090 RC1141   MLKSSKKEDSSKKNQNNKLIFTVRKLFSPIKNFFRKTKTPDNFFGVIKRLKINSQKMTLD 
C112RRA0006 tlyC; Hemolysin C   MLKSSKKEDSSKKNQNNKLIFTVRKLFSPIKNFFRKTKTPDNFFDVIKRLKINSQKMTLD 

                                         70        80        90       100       110       120        
                                ....|....|....|....|....|....|....|....|....|....|....|....|
rco_ORF1032 2890083090 RC1141   ERNILANLLELEDKTIEDIMVPRSDIAAIKLTTNLEELSESIKLEVPHTRTLIYDGTLDN 
C112RRA0006 tlyC; Hemolysin C   ERNILANLLELEDKTIEDIMIPRSDIAAIKLTTNLEELSESIKLEVPHTRTLIYDGTLDN 

                                        130       140       150       160       170       180     
                                ....|....|....|....|....|....|....|....|....|....|....|....|
rco_ORF1032 2890083090 RC1141   VVGFIHIKDLFKALATKQNGRLKKLIRKHIIAAPSMKLLDLLAKMRRERTHIAIVVDEYG 
C112RRA0006 tlyC; Hemolysin C   VVGFIHIKDLFKALATKQNGRLKKLIRKHIIAAPSMKLLDLLAKMRRERTHIAIVVDEYG 

                                        190       200       210       220       230       240     
                                ....|....|....|....|....|....|....|....|....|....|....|....|
rco_ORF1032 2890083090 RC1141   GTDGLVTIEDLIEEIVGRIDDEHDQQLDSDNFKVINNSTIIANARVEVEVLEEIIGEKLH 
C112RRA0006 tlyC; Hemolysin C   GTDGLVTIEDLIEAIVGRIDDEHDQQLDSDNFKVINNSTIISNARVEVEVLEEIIGEKLQ 

                                        250       260       270       280       290          
                                ....|....|....|....|....|....|....|....|....|....|....|....
rco_ORF1032 2890083090 RC1141   NDYDEFDTIGGLVLTRVSSVPAIGTRIDISENIEIEVTDATPRSLKQVKIRLKNGLNGQ 
C112RRA0006 tlyC; Hemolysin C   NDDDEFDTIGGLVLTRVSSVPAIGTRIDISANIEIEVTDATPRSLKQVKIRLKNGLNGK 
