# Supplementary material for: RickA Expression Is Not Sufficient to Promote Actin-Based Motility of Rickettsia raoultii
Source: PLoS One. 2008 Jul 9;3(7):e2582. doi: 10.1371/journal.pone.0002582 (PMC2440523; doi:10.1371/journal.pone.0002582)
Supplement: Table S1 — Oligonucleotide primers used for PCR amplification and sequencing of rickA. (0.03 MB DOC) [file pone.0002582.s004.doc]

**Table S1. Oligonucleotide primers used for PCR amplification and sequencing of *rickA***

| **primer** | **nucleotide sequence** | **gene position relative**  **to the *R. conorii* ORF** |
| --- | --- | --- |
| RIC-30 F^1^ | TTGCTTTTGTACAAGAAATTT | -30-51 |
| RIC-30 F^1^ | TTGCTTTTGTACAAGAAATTT | -30-51 |
| RIC486 F^2^ | CTTACAATTCATTAATGCTGAAG | 486-509 |
| RIC1200 F^2^ | GGCAAAATGTTAAAAATGTTT | 670-691 |
| RIC1400 F^2^ | AGTTACAAAAGATAGTAAGTAA | 859-881 |
| RIC500 R^2^ | AATTGTAAGGGATTTGTCATAT | 472-494 |
| RIC700 R^2^ | TTGTTCATCAATCTTTCT | 750-768 |
| RIC+430 R^2^ | CCRGYTTTTTAACCGTAGTAG | 1180-1201 |
| RIC+740R^1^ | CCCGCTTCCGCGGGAATGACAT | +11-+32 |

(1) Primers used for PCR amplification, (2) Primers used for sequencing
